# Risk factors associated with acute kidney injury in a cohort of hospitalized patients with COVID-19

**DOI:** 10.1186/s12882-023-03172-8

**Published:** 2023-05-22

**Authors:** Kateir Contreras-Villamizar, Oscar Barbosa, Ana Cecilia Muñoz, Juan Sebastián Suárez, Camilo A. González, Diana Carolina Vargas, Martha Patricia Rodríguez-Sánchez, Paola García-Padilla, Martha Carolina Valderrama-Rios, Jorge Alberto Cortés

**Affiliations:** 1 Nephrology Unit, Kr 7 40 62 Hospital Universitario San Ignacio, 110231 Bogotá, DC Colombia; 2 Department of Internal Medicine, School of Medicine, Pontificia Universidad Javeriana, Bogotá, DC Colombia; 3 Department of Internal Medicine, School of Medicine, Universidad Nacional de Colombia, Bogotá Campus, Bogotá, DC Colombia; 4 Infectology Unit, Hospital Universitario Nacional de Colombia, Bogotá, DC Colombia

**Keywords:** Acute Kidney Injury, COVID-19, Risk factors, Outcomes

## Abstract

**Background:**

Patients with COVID-19 have a high incidence of acute kidney injury (AKI), which is associated with mortality. The objective of the study was to determine the factors associated with AKI in patients with COVID-19.

**Methodology:**

A retrospective cohort was established in two university hospitals in Bogotá, Colombia. Adults hospitalized for more than 48 h from March 6, 2020, to March 31, 2021, with confirmed COVID-19 were included. The main outcome was to determine the factors associated with AKI in patients with COVID-19 and the secondary outcome was estimate the incidence of AKI during the 28 days following hospital admission.

**Results:**

A total of 1584 patients were included: 60.4% were men, 738 (46.5%) developed AKI, 23.6% were classified as KDIGO 3, and 11.1% had renal replacement therapy. The risk factors for developing AKI during hospitalization were male sex (OR 2.28, 95% CI 1.73–2.99), age (OR 1.02, 95% CI 1.01–1.03), history of chronic kidney disease (CKD) (OR 3.61, 95% CI 2.03–6.42), High Blood Pressure (HBP) (OR 6.51, 95% CI 2.10–20.2), higher qSOFA score to the admission (OR 1.4, 95% CI 1.14–1.71), the use of vancomycin (OR 1.57, 95% CI 1.05–2.37), piperacillin/tazobactam (OR 1.67, 95% CI 1.2–2.31), and vasopressor support (CI 2.39, 95% CI 1.53–3.74). The gross hospital mortality for AKI was 45.5% versus 11.7% without AKI.

**Conclusions:**

This cohort showed that male sex, age, history of HBP and CKD, presentation with elevated qSOFA, in-hospital use of nephrotoxic drugs and the requirement for vasopressor support were the main risk factors for developing AKI in patients hospitalized for COVID-19.

**Supplementary Information:**

The online version contains supplementary material available at 10.1186/s12882-023-03172-8.

## Background

Infection by the severe acute respiratory syndrome coronavirus 2 (SARS CoV-2) has been responsible for the coronavirus disease 2019 (COVID-19) pandemic that began at the end of 2019 and continues to the present, leading to high rates of morbidity and mortality and a global public health crisis [1]. The number of infections has been increasing, exceeding 530 million worldwide, with more than 6 million deaths [2].

Patients requiring admission to an intensive care unit (ICU), mechanical ventilation and renal replacement therapy (RRT) have high mortality rates [3, 4]. The frequency of AKI has varied across studies, depending on geographic location; in China, at the beginning of the pandemic, it was no greater than 30% [5, 6]. However, studies in the United States, Spain, France and Colombia showed frequencies up to 80% [7–10].

In cases of severe AKI, mortality was greater than 50%, often related to multiorgan involvement. Between 5 and 15% of cases required RRT, and this was the factor with the worst prognosis, being associated with mortality rates greater than 90% [7, 11, 12]. Some risk factors for AKI have been proposed in patients with COVID-19; however, information is scarce, and knowing these factors will allow early intervention to define patients at risk and intervene in a timely manner to organize care networks and optimize resources.

The objective of this study was to establish the risk factors associated with the appearance of AKI in adult patients hospitalized with COVID-19 in a cohort in two university hospitals in Bogotá, Colombia.

## Materials and methods

### Study design

A retrospective cohort study was conducted in two institutions in Bogotá, DC: The National University Hospital of Colombia and the San Ignacio University Hospital, which are level 4 hospitals that care for adult patients from the city of Bogotá and other regions in Colombia (a population of more than 8 million inhabitants). During the pandemic peaks, hospitals had approximately 150 intensive care beds and 100 hospital care beds for COVID-19. The patients included were hospitalized from March 1, 2020, to March 31, 2021, during the first two pandemic waves in Colombia.

### Patient selection

Patients older than 18 years with SARS-CoV-2 infection confirmed by reverse transcription polymerase chain reaction (RT-PCR) or viral antigen were included. Patients who were hospitalized for less than 48 h in the participating institutions, hospitalized more than 72 h at the remission hospital, with less than two creatinine measurements during the stay, pregnant women, and patients with stage 5 chronic kidney disease (CKD), solid organ transplant, or obstructive uropathy were excluded.

### Sample

A sample size calculation was performed based-on 20% incidence of AKI associated with COVID-19 [13], a statistical power of 80%, an alpha of 0.05, and odds ratio (OR) of association for age of 1.6 [14]. The size was estimated using Open Epi® [15] with a required sample of 1626 patients, which exceeds the estimate of 10 to 15 AKI cases per association variable.

### Data collection

A clinical research form was created in the REDCap® platform. The data were collected from a review of the medical records, and the data of each variable were extracted and recorded in REDCap®. A data quality review was performed by a researcher. Laboratory information was used to detect patients with at least two values of serum creatinine, with which the identification of AKI was performed. Subsequently, the KDIGO classification was performed [16] and a final review was made by the experts to verify that the definition and classification were correct.

### Definitions

Hospital-acquired AKI was defined as an increase or decrease in creatinine greater than or equal to 0.3 mg/dl with respect to baseline creatinine according to KDIGO in a period of 48 h to 7 days during the 28 days following hospital admission [16]. Baseline creatinine was considered if it was available in the different computer systems consulted (defined as the measurement in the three months prior to admission) [17, 18]. Urinary volume was not considered given the low frequency of recording these data in the clinical history. Immunosuppression was defined as a history of solid neoplasia, hematologic neoplasia, HIV infection, systemic lupus erythematosus, or ANCA-associated vasculitis. Leukocytosis was defined as a leukocyte count greater than 12 × 10 ^ 3/μl and lymphopenia as a lymphocyte count less than 0.8 × 10 ^ 3/μl. HBP, diabetes mellitus (DM), CKD and heart failure were recorded considering whether the patient had a history of this disease recorded in the medical history of hospitalization. Individuals were considered obese or overweight based on a personal medical history of these conditions, or based on their BMI, weight and height data recorded in the medical record: individuals with a BMI of 25–29.9 kg/m^2^ were deemed overweight, whereas those with a BMI ≥ 30 kg/m^2^ were considered obese.

### Ethical aspects

The project was approved by the Research and Ethics Committee (REC) of each of the participating institutions. Approval CEI-2021–05-05 of the National University Hospital and CEI -0631–21 of the San Ignacio University Hospital. Individual informed consent was waived by the approving (REC) in both institutions, as permitted by the fore-mentioned regulation, considering the retrospective nature of the data. Patient information was processed anonymously.

### Statistical analysis

A descriptive analysis of the sociodemographic and clinical characteristics of the study population was performed. Categorical variables are expressed as absolute number with percentage. Continuous variables are expressed as mean with standard deviation (SD) or median with interquartile range (IQR) where appropriate.

Bivariate analyses were performed to evaluate the relationship between the variables identified in the literature and those potentially related by biological plausibility with the AKI outcome, using the Chi-squared test for categorical variables, and Student’s t-test or Wilcoxon rank test for continuous variables. For the analysis to determine association with the outcome adjusting for potential confounders, a multivariate logistic regression model was performed, in which the outcome variable was the appearance of AKI. Variables that had a value of *p* < 0.2 and those of clinical relevance were included in the model, such as the clinically relevant and previously defined interactions: 1. Previous treatment with antihypertensive drugs and diuretics; 2. PaO_2_/FiO_2_ (PAFI) and invasive mechanical ventilation; 3. Arterial hypertension and previous treatment with antihypertensives; 4. Heart failure and previous treatment with antihypertensives, 5. Heart failure and previous treatment with diuretics. Variables with missing data greater than 30% were excluded from the analysis in the logistic multivariable regression model. The stepwise technique using the statistical significance of each independent variable, was carried out for model selection. Analysis of measures of fit for each model, including Akaike's Information Criterion (AIC), Bayesian Information Criterion (BIC), and McFadden's adjusted R2, was performed. The model with the greatest parsimony and lowest error was chosen. The analysis was performed with the statistical package Stata® (see 15.0, StataCorp, Texas, USA).

## Results

**Fig. 1:**
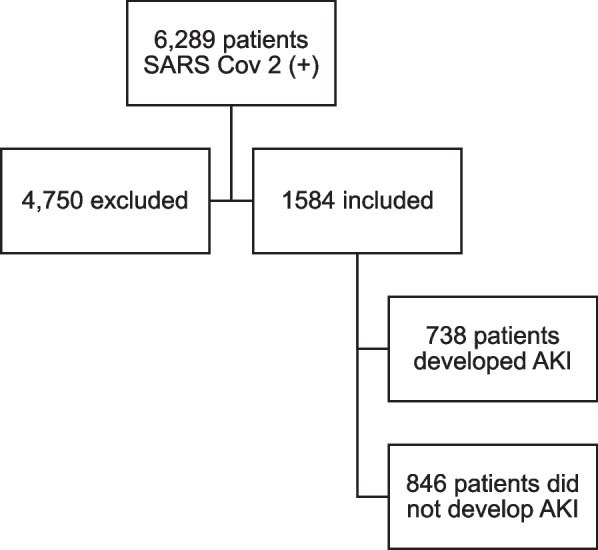
Flowchart of patients in the study

A total of 1584 patients were included (Fig. 1), of which 956 (60.4%) were male, the median age was 63 years old (IQR 21.5), 664 (41.9%) and had a history of HBP, 403 (25.4%) of smoking and 325 (20.5%) of diabetes mellitus (DM). At admission, 1309 (82.6%) had respiratory symptoms, 443 (28%) had gastrointestinal symptoms, and 82 (5.2%) were asymptomatic. The information discriminated by groups is shown in Table 1.

**Table 1:** Initial characteristics

Characteristic	No AKI (*n* = 846)	AKI (*n* = 738)	*p*-value
Age, median in years (IQR)	61 (23)	66 (19)	< 0.001
Gender—males, n (%)	436 (51.5)	520 (70.5)	< 0.001
Days from symptoms onset to admission, median in days (IQR)	7 (6)	6 (4)	0.102
Overweight or obesity, n (%)	178 (21)	215 (29.1)	< 0.001
Tobacco use, n (%)	188 (22.2)	215 (29.1)	0.002
Diabetes mellitus, n (%)	152 (18)	173 (23.4)	0.007
High blood pressure, n (%)	299 (35.3)	365 (49.5)	< 0.001
Chronic kidney disease, n (%)	22 (2.6)	79 (10.7)	< 0.001
Heart failure, n (%)	37 (4.4)	69 (9.4)	< 0.001
Charlson comorbidity index, n (%)			
Low comorbidity (CCI ≤ 2)	797 (94.2)	648 (87.8)	
High comorbidity (CCI ≥ 3)	49 (5.8)	90 (12.2)	
Immunosuppression, n (%)	4 (0.5)	5 (0.7)	0.589
Previous treatment			< 0.001
Immunosupressive, n (%)	19 (2.3)	16 (2.2)	0.916
Antihipertensives, n (%)	622 (73.5)	506 (68.6)	0.030
Non IACE/ARAII antihipertensives, n (%)	96 (11.4)	151 (20.5)	< 0.001
Statins, n (%)	542 (64.1)	406 (55)	< 0.001
Diuretics, n (%)	517 (61.1)	365 (49.5)	< 0.001
NSAIDs, n (%)	26 (3.1)	48 (6.5)	0.001
Body temperature at admission, median in degrees Celsius (IQR)	36.7 (1)	36.6 (0.7)	0.014
Fever at admission, n (%)	101 (12.2)	81 (11.5)	0.682
Sistolic arterial pressure at admission, median in mmHg (IQR)	123 (25)	123 (29.5)	0.059
Diastolic arterial pressure at admission, median in mmHg (IQR)	75 (14)	74 (17)	0.001
Blood oxygen saturation at admission, median in % (IQR)	88 (8)	88 (10)	0.408
qSOFA at admission, n (%)			< 0.001
qSOFA 0	385 (45.5)	269 (26.5)	
qSOFA ≥ 1	461 (54.5)	469 (63.5)	
PaFi at admission, median (IQR)	262 (106)	217 (159)	< 0.001
PAFI by severity, n (%)			< 0.001
PaFi ≥ 300	653 (77.2)	428 (58)	
PaFi 101—299	140 (16.6)	182 (24.7)	
PaFi ≤ 100	53 (6.3)	128 (17.3)	
Lymphocyte count at admission, median in cells × 10^3/ul (IQR)	1.1 (0.7)	0.8 (0.7)	< 0.001
C-reactive protein at admission, median in mg/l (IQR)	91.7 (119.9)	128.4 (135.4)	< 0.001
Creatinine at admission, median in mg/dl (IQR)	0.8 (0.3)	1.1 (0.4)	< 0.001
Blood ureic nitrogen at admission, median in mg/dl (IQR)	15.5 (7.5)	24.2 (18.3)	< 0.001
Sodium at admission, median in mmol/l (IQR)	137 (5)	137 (6.7)	0.296
Lactate dehydrogenase at admission, median in U/l (IQR)	325.5 (164.1)	386.5 (250.4)	< 0.001
D-dimer at admission, median in ng/ml (IQR)	776 (931)	1,055 (1,561)	< 0.001
Platelets al admission, median in cells × 10^3/ul (IQR)	226 (113.9)	211 (118)	< 0.001

*AKI* Acute kidney injury, *IQR* Interquartile range, *CCI* Charlson Comorbidity Index, *IACE* Inhibitor of the angiotensin-converter enzyme, *ARAII* Angiotensin II receptor antagonist, *NSAID* non-steroid anti-inflammatory drug, *mmHg* Millimeters of mercury, *qSOFA* Quick sequential organ failure assessment, *PaFi* PaO2/FiO2

AKI was confirmed in 738 patients (46.5%), and the severity distribution according to KDIGO was 448 (60.7%), 116 (15.7%), and 174 (23.6%) for stages 1, 2 and 3, respectively. The information discriminated by center is shown in Table 2.

**Table 2:** Incidence and severity of acute kidney injury

	Centre A (*n* = 1102)	Centre B (*n* = 482)
Incidence AKI	453 (41.1%)	285 (59.1)
KDIGO 1	309 (28.0%)	139 (28.8%)
KDIGO 2	74 (6.72%)	42 (8.71%)
KDIGO 3	70 (6.35%)	104 (21.5%)
Mortality in AKI	178 (39.2%)	158 (55.4%)

*AKI* Acute Kidney Injury, *KDIGO* Kidney Disease Improving Global Outcome

### Differences between groups according to the development of AKI

Among the subgroups, it was more common to find older patients with AKI, as well as those with overweight or obesity, smoking, arterial hypertension, diabetes mellitus, CKD and heart failure. Those who developed AKI had greater comorbidity (Charlson greater than or equal to 3), as well as quick Sepsis-related Organ Failure Assessment qSOFA greater than 1, compared to the group that did not present AKI. Table 1 shows the initial characteristics of the evaluated cohort and the differences between the two groups.

Patients with AKI associated with COVID-19 had greater exposure to nephrotoxic potentials, such as nonsteroidal anti-inflammatory drugs (NSAIDs), vancomycin, piperacillin/tazobactam, propofol and contrast media. One hundred seventy-two patients (14.1%) received the combination of antibiotics piperacillin/tazobactam plus vancomycin. No statistically significant difference was found in the frequency of treatment with amikacin or steroids during hospitalization when compared with the group that did not undergo AKI (data not shown).

### Outcomes

Among patients with AKI, 11.1% required RRT, the most used modality was continuous, were more frequently admitted to the ICU and needed vasopressor support and invasive mechanical ventilation. The average number of days of hospitalization in the general ward was higher in this group, as were infections acquired during hospitalization, the majority of which were infections of the bloodstream, urinary tract, and pneumonia. Hospital mortality for the AKI group was 336 (45.5%) vs. 99 (11.7%) for the group without AKI (*p* < 0.0001). Table 3 summarizes the hospital outcomes.

**Table 3:** In-hospital outcomes

Characteristics	Total (*n* = 1,584)	No AKI (*n* = 846)	AKI (*n* = 738)	*p*-value
Vasopressor support during hospitalization—n (%)	545 (34.3)	129 (15.3)	416 (56.4)	< 0.001
Mechanical ventilation during hospitalization—n (%)	620 (39.1)	170 (20.1)	450 (61)	< 0.001
ICU admission—n (%)	690 (43.6)	216 (25.5)	474 (64.2)	< 0.001
Mean hospitalization—days (IQR)	10 (12)	8 (9)	14 (15)	< 0.001
Renal replacement therapy modalities n (%)			82 (11.1%)	
Peritoneal dialysis			1 (1.2%)	
Intermittent hemodialysis			27 (32.9%)	
CRRT			54 (65.8%)	
Infection acquired during hospitalization—n (%)	263 (16.6)	74 (8.7)	189 (25.6)	< 0.001
Bloodstream infection—n (%)	49 (3.1)	15 (1.8)	34 (4.6)	< 0.001
Urinary tract infection—n (%)	41 (2.6)	12 (1.4)	29 (3.9)	0.002
Healthcare-associated pneumonia—n (%)	80 (5.1)	18 (2.1)	62 (8.4)	< 0.001
Infection of the operative site—n (%)	1 (0.06)	0 (0)	1 (0.14)	0.284
Another infection during hospitalization—n (%)	92 (5.8)	29 (3.4)	63 (8.5)	< 0.001
Intrahospital mortality—n (%)	435 (27.5)	99 (11.7)	336 (45.5)	< 0.001

*AKI* Acute kidney injury, *ICU* Intensive care unit, *CRRT* Continuous Renal replacement therapy

### Risk factors for AKI

In the multivariate analysis, the variables with statistical significance that were associated with an increased risk of AKI were sex, age, HBP, CKD, qSOFA, treatment with vancomycin, piperacillin tazobactam, and requirement of vasopressor support. The PAFI interaction and invasive mechanical ventilation (Interaction 2) predicted the appearance of AKI, and in contrast, treatment with statins and the interaction of arterial hypertension and previous treatment with antihypertensives (Interaction 3) were protective factors. Table 4 shows the variables associated with AKI in the final model.

**Table 4:** Risk factors for acute kidney injury for in-hospital patients with COVID-19. Adjusted model

Variable	Adjusted OR	95% CI
Sex	2.28	1.73—2.99
Age	1.02	1.01—1.03
Overweight or obesity	1.31	0.98—1.75
History of HBP	6.51	2.10—20.20
History of CKD	3.61	2.03—6.42
Previous Treatment with statins	0.57	0.38—0.85
Higher qSOFA score	1.4	1.14—1.71
Lower platelet count at admission	1.01	0.99—1.02
Higher CRP at admission	1	0.99—1.01
Higher D-dimer at admission	1	0.99—1.01
Treatment with vancomycin	1.57	1.05—2.37
Treatment with Piperacillin tazobactam	1.67	1.20—2.31
Treatment with Propofol	1.45	0.94—2.24
Vasopressor support	2.39	1.53—3.74
Interaction # 2^a^	1.3	1.05—1.61
Interaction # 3^b^	0.36	0.14—0.94

*CI* Confidence interval, *HBP* High blood pressure, *CKD* chronic kidney disease, *qSOFA* Quick sequential organ failure assessment, *CRP* C-reactive protein, *PaFi* PaO2/FiO2

^a^PAFI and invasive mechanical ventilation

^b^Arterial hypertension and previous treatment with antihypertensives

## Discussion

In this cohort of 1584 patients hospitalized with COVID-19, conducted in a middle-income country, the incidence of AKI was 46.5%, similar to that reported in other American cohorts [19]. The risk factors for AKI in this scenario were male gender, age, having comorbidities such as hypertension and CKD, onset with qSOFA greater than 1, receiving inpatient treatment with vancomycin or piperacillin tazobactam and the use of vasopressor support, findings consistent with what has been published recently in other cohorts [7–9, 20, 21]. As protective factors, female gender, previous use of statins, and interaction with hypertension and previous treatment with antihypertensives were found.

AKI is a clinically important entity due to its impact on costs, morbidity and mortality, findings that are clearly described and documented in critically ill patients [22] that are increasingly more robust in patients with COVID-19 [19, 23].

Obesity and smoking did not reach statistical significance as determinant risk factors for the appearance of AKI lesions, as has been documented in other studies [23]. In this cohort, the obesity variable had a high rate of data loss, which can affect the results and explain this finding.

Regarding the severity of AKI found in our population, we explained that most AKI cases are KDIGO 1 because most patients did not have comorbidities, nor were they critically ill. However, the patients in whom the disease advanced to severe forms ended up developing severe AKI up to KDIGO 3, with very few reaching only AKI KDIGO 2. We also found that most people consulted the first week after the onset of symptoms, facilitating early initiation of treatment, including nephroprotection measures.

The length of hospital stay, requirement of vasopressor support and mechanical ventilation were higher in the AKI subgroup, as were infections associated with health care, which increases the requirement for the use of antibiotics with nephrotoxic potential, such as vancomycin and piperacillin tazobactam [24]. Higher qSOFA scores, lower PAFI values, elevated levels of D-dimer and CRP, and low platelet counts were found, findings that reaffirm what has been documented in other studies that patients with AKI and COVID-19 are more inflamed. No difference was found in the previous use of immunosuppressive treatment or in the history of immunosuppressive conditions, which is like previous studies [7–9, 18–20, 24, 25].

The timing and modality of RRT was at the discretion of the nephrologist. CRRT was the most used, under indication of hemodynamic instability, in accordance with the large proportion of vasopressor use reported in the subgroup with AKI.

This study has the usual limitations of a retrospective cohort, which can generate biases. Urinary output was not considered for the diagnosis of AKI, which could underestimate the incidence of the condition; however, this is a limitation in real life given the underreporting of this variable. The low frequency of recording weight and height led to the loss of the obesity variable, which is a known risk factor for severity in COVID-19. We had fewer patients than initially calculated, we run into the risk that this study would not have enough power to find differences between the groups; however, we had the advantage of finding a higher AKI frequency, which was more than double that used for the calculation of the sample size. Therefore, the smaller number of patients included does not generate limitations.

As strengths, the inclusion of two university institutions with a diverse population and a larger sample size than other previously published cohorts are highlighted.

In conclusion, AKI is a frequent clinical entity in patients hospitalized for COVID-19 in Colombia. Male sex, age, history of HBP and CKD, presentation with elevated qSOFA and requirement of vasopressor support and exposure to piperacillin, tazobactam and vancomycin were the main risk factors. Identifying patients with greater AKI allows for early introduction of nephroprotection measures, such as limiting the use of nephrotoxins and preventing or mitigating the compromise of renal function. This identification of risk can contribute to times of contingency to refer these patients to more complex centers to provide timely care.

## Supplementary Information

**Additional file 1: Supplement 1.** Definitions of the risk factors for acute kidney injury for in-hospital patients with COVID-19 - adjusted model.

## Data Availability

The datasets generated during this study are available from the corresponding author on reasonable request via email.

## Abbreviations

AIC: Akaike's Information Criterion
AKI: Acute kidney injury
ANCA: Antineutrophil cytoplasmic antibodies
ARAII: Angiotensin II receptor antagonist
BIC: Bayesian Information Criterion
BMI: Body mass index
CCI: Charlson comorbidity index
CKD: Chronic kidney disease
COVID 19: Coronavirus disease 2019
CRRT: Continuous renal replacement therapy
CRP: C-reactive protein
DM: Diabetes mellitus
HBP: High Blood Pressure
HIV: Human immunodeficiency virus
IACE: Inhibitor of the angiotensin-converter enzyme.
ICU: Intensive care unit
IQR: Interquartile rang
NSAID: Nonsteroidal anti-inflammatory drugs
OR: Odds ratio
PD: Peritoneal dialysis
qSOFA: Quick Sepsis-related Organ Failure Assessment
RRT: Renal replacement therapy
PaFi: PaO2/FiO2
RT-PCR: Reverse transcription polymerase chain reaction
SARS—CoV2: Severe acute respiratory syndrome coronavirus 2
SD: Standard deviation
