# Supplementary material for: Risk factors associated with acute kidney injury in a cohort of hospitalized patients with COVID-19
Source: BMC Nephrol. 2023 May 22;24:140. doi: 10.1186/s12882-023-03172-8 (PMC10201026; doi:10.1186/s12882-023-03172-8)
Supplement: Supplementary file 1 — Additional file 1: Supplement 1. Definitions of the risk factors for acute kidney injury for in-hospital patients with COVID-19 - adjusted model. [file 12882_2023_3172_MOESM1_ESM.docx]

**Supplement 1 - Definitions of the risk factors for acute kidney injury for in-hospital patients with COVID-19 - adjusted model**

- Sex: Biological sex at birth, and corresponds to Female or Male
- Age: years of age at the time of entry into the registry, calculated from the date of birth
- Overweight or obesity: history of overweight or obesity, as part of the diagnoses of that hospitalization, or described on physical examination, according to the BMI, overweight is 25-29.9 kg/m2, and obesity is ≥30 kg/m2.
- History of HBP: history of arterial hypertension referred in the clinical history, by the patient or a relative
- History of CKD: history of CKD referred in the clinical history, by the patient or a relative
- Previous treatment with statins: history of statins referred in the clinical history, by the patient or a relative
- qSOFA score: corresponds to qSOFA score at admission, consisting of three items: respiratory rate (RR) ≥ 22 breaths per minute, altered mentation (Glasgow Coma Scale [GCS] < 15), and systolic blood pressure (SBP) < 100 mmHg.
- Platelet count at admission: platelet level at admission in the units cels/ul
- Higher CRP at admission: CRP at admission: Initial c-reactive protein measurement result, in the units mg/l
- Higher D-dimer at admission:
- D-dimer at admission: Initial d-dimer measurement result, in the units mg/l
- Treatment with vancomycin: Vancomycin administration during hospitalization
- Treatment with piperacillin/tazobactam: Administration of piperacillin-tazobactam during hospitalization
- Treatment with propofol: Propofol administration during hospitalization
- Vasopressor support: requirement of vasopressor support during hospitalization, evaluating the record in the clinical history of formulation of norepinephrine (noradrenaline) or epinephrine (adrenaline) or vasopressin or dopamine.
- PAFI: Ratio of arterial oxygen pressure and fraction of inspired oxygen (PaO2/ FIO2) in initial arterial blood gases.
- Invasive mechanical ventilation: record of mechanical ventilation requirement during hospitalization
